# Retraction: Physio-anatomical modifications and elemental allocation pattern in *Acanthus ilicifolius* L. subjected to zinc stress

**DOI:** 10.1371/journal.pone.0296156

**Published:** 2023-12-14

**Authors:** The PLOS ONE Editors

The *PLOS ONE* Editors retract this article [1] because it was identified as one of a series of submissions for which we have data concerns and concerns about authorship, competing interests, and peer review. We regret that the issues were not addressed prior to the article’s publication.

In addition, the following concerns were raised:

- The following images appear similar:
  ○ Fig 1A Control of [1] and Fig 1A of [2]
  ○ Fig 1B Control of [1] and Fig 2A of [2]
  ○ Fig 4 of [1] and Fig 7A of [2]
- The individual level data underlying the study have not been made available in accordance with the PLOS Data Availability Policy.

The authors explain that the studies were conducted simultaneously and that the panels are the same as they represent the same controls. They provided some individual level data to support the article, but some of the individual level data for Table 1 and 2 are no longer available. In our editorial assessment of the data provided for Table 1, we identified standard error value discrepancies between the dataset and the published table. The corresponding author’s explanations did not resolve these concerns.

Figs 1 and 4 report material from [2], published in 2019 by Taylor & Francis, which are not offered under a CC BY license and are therefore excluded from this article’s [1] license. At the time of retraction, the article [1] was republished to note this exclusion in the Figs 1 and 4 legends and the article’s copyright statement.

All authors disagreed with the retraction decision.
